# Deep Learning-Enhanced Sampling-Based Path Planning for LTL Mission Specifications

**DOI:** 10.3390/s24102998

**Published:** 2024-05-09

**Authors:** Changmin Baek, Kyunghoon Cho

**Affiliations:** Department of Information and Telecommunication Engineering, Incheon National University, Incheon 22012, Republic of Korea; 1901681@inu.ac.kr

**Keywords:** deep learning-based control synthesis, formal methods, mission-based path planning, sampling-based path planning

## Abstract

The presented paper introduces a novel path planning algorithm designed for generating low-cost trajectories that fulfill mission requirements expressed in Linear Temporal Logic (LTL). The proposed algorithm is particularly effective in environments where cost functions encompass the entire configuration space. A core contribution of this paper is the presentation of a refined approach to sampling-based path planning algorithms that aligns with the specified mission objectives. This enhancement is achieved through a multi-layered framework approach, enabling a simplified discrete abstraction without relying on mesh decomposition. This abstraction is especially beneficial in complex or high-dimensional environments where mesh decomposition is challenging. The discrete abstraction effectively guides the sampling process, influencing the selection of vertices for extension and target points for steering in each iteration. To further improve efficiency, the algorithm incorporates a deep learning-based extension, utilizing training data to accurately model the optimal trajectory distribution between two points. The effectiveness of the proposed method is demonstrated through simulated tests, which highlight its ability to identify low-cost trajectories that meet specific mission criteria. Comparative analyses also confirm the superiority of the proposed method compared to existing methods.

## 1. Introduction

Path planning is a critical element in robotics, evolving from its initial focus on straightforward two-dimensional tasks to addressing more complex challenges involving advanced robotic systems. This evolution is well-documented in the research literature, including studies such as [1,2,3,4,5,6,7,8,9], showcasing the field’s shift towards intricate scenarios like those involving robot manipulators.

Today, path planning goes beyond simple obstacle navigation to involve robots in multifaceted missions, which might include tasks like area coverage or sequential target visits. These complex missions require sophisticated algorithms that not only plan paths but also coordinate multi-robot operations, as discussed in [10,11,12,13,14].

In the realm of mission-driven path planning, the process often begins with defining the mission type, typically focusing on coverage. Comprehensive algorithms are then developed to integrate these mission specifications effectively with path planning strategies. A significant challenge is converting missions, often described in natural language, into forms that are compatible with path planning algorithms. This necessity has led to the increased use of formal methods like Linear Temporal Logic (LTL), Computation Tree Logic (CTL), and μ-calculus to precisely define robot mission requirements [15,16,17]. LTL, in particular, has become prominent for its ability to clearly articulate robotic objectives [18,19,20,21]. For instance, LTL formulas like ϕ=◊(A)∧◊(B) explicitly denote tasks such as delivering items to region (*A*) and collecting items from region (*B*). This specification showcases the utility of LTL in formulating simple yet explicit mission objectives. Furthermore, the flexibility of LTL allows for the expression of more complex tasks by combining various operators, thereby accommodating a broader range of mission requirements and enhancing the adaptability of robotic systems to diverse operational contexts.

Moreover, path planning is increasingly tasked with not just finding feasible paths but also optimizing them in terms of various cost considerations. For example, optimizing a robot’s trajectory to maintain strong communication in varying signal environments illustrates the importance of cost-effective routing, where the optimal path minimizes time in low-signal areas. This scenario highlights the ongoing challenge of balancing cost efficiency with complex mission fulfillment, especially when integrating sophisticated mission specifications like LTL formulas into dynamic environments.

Computational efficiency is another critical aspect of path planning. Sampling-based motion planning methods, which are prevalent in practical applications, often require extensive sampling to find viable solutions. These methods, marked by thorough exploration of the state space, demand numerous samples, particularly in low-cost path search algorithms like ${\mathrm{RRT}}^{*}$. The complexity of state spaces and collision constraints adds significant computational burden to the sampling process. To enhance planning efficiency, machine-learning techniques have been incorporated into sampling-based motion planning algorithms [22,23,24]. These approaches utilize data-driven learning to reduce redundant sampling, thereby facilitating the more efficient discovery of solution paths.

This paper addresses the challenge of developing cost-aware path planning strategies within the framework of dynamic constraints and Linear Temporal Logic (LTL) specifications. Our primary contribution lies in enhancing the efficiency of the search process to identify low-cost trajectories that not only fulfill LTL mission specifications but also optimize quantitative performance metrics across a continuous configuration space. The use of LTL allows us to enforce qualitative mission requirements, ensuring that all solutions adhere to specified behavioral constraints. Simultaneously, our optimization techniques focus on minimizing tangible costs such as travel distance, time, or energy, thereby integrating qualitative mission goals with quantitative optimization objectives. This dual approach ensures that our path planning solutions are both compliant with mission-specific requirements and optimized for operational efficiency.

We employ syntactically co-safe LTL formulas to define mission objectives, calculating trajectory costs as an aggregate of expenses incurred throughout the entire path. To address this complex challenge, we utilize a sampling-based path planning methodology that integrates specified LTL conditions into the search tree expansion, consistently aligning the process with the LTL-driven mission.

**Figure 1:**
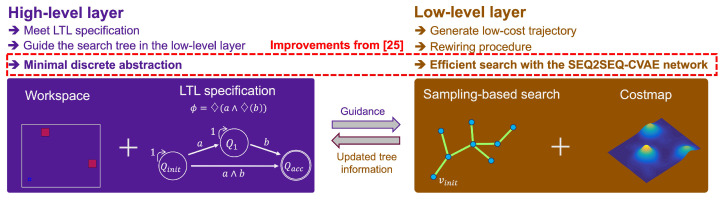
An overview of the proposed method, illustrating the interplay between the high-level layer that meets LTL specifications and the low-level layer that searches for low-cost trajectories, underscored by enhancements from our previous work [25].

Building upon the foundation laid in our previous research [25], we have further refined the search tree expansion method, as illustrated in Figure 1. Maintaining the dual-layered structure from [25], our strategy employs high-level and low-level layers to ascertain solution trajectories that meet both LTL specifications and cost-optimization criteria. The high-level layer ensures trajectory compliance with LTL specifications by directing the search in the low-level layer, optimized to discover low-cost pathways through a rewiring procedure inspired by ${\mathrm{RRT}}^{*}$ [26]. This setup facilitates the continuous refinement of tree information for ongoing adjustments.

The innovation in our methodology manifests in two major improvements over [25]: the adoption of a minimal abstraction approach in the high-level layer, eliminating the need for mesh decomposition, and the integration of a meticulously fine-tuned SEQ2SEQ-CVAE deep neural network. This network significantly enhances the efficiency of identifying low-cost trajectories, marking a substantial advancement in robotic path planning.

Furthermore, to address the necessity of treating the path planning problem as an integrated task rather than separate subtasks, we consider the interdependencies and dynamics of the trajectory between points. By optimizing the path in an integrated manner, we ensure that constraints such as cost, time, and the sequence of operations are holistically considered. This often leads to solutions that are not only globally optimal but also more robust to changes in the environment. This approach is particularly advantageous in real-world applications where conditions may change unpredictably, requiring a flexible and adaptive path planning strategy. The integrated method facilitates real-time adjustments and optimizations, critical in dynamic settings where separate optimization of subtasks might fail to account for evolving operational contexts.

**Figure 2:**
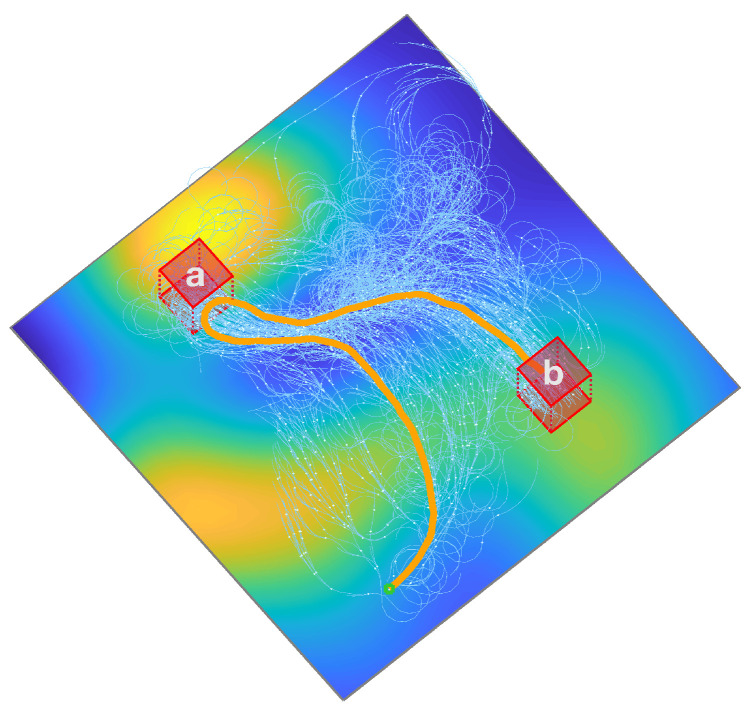
An illustrative trajectory generated by the proposed method within the test scenario. The search tree, visualized in orange, strategically guides the trajectory through areas of lower cost, as indicated by the blue regions, while adhering to the specified LTL formula ϕ=◊(a∧◊(b)).

Figure 2 illustrates a solution trajectory generated by our proposed method in a simulated test environment. This trajectory is designed to comply with the requirements of the Linear Temporal Logic (LTL) formula ϕ=◊(a∧◊(b)), which dictates a sequential visit to specified regions *a* and *b*. The method dynamically expands the search tree to discover an optimal path that satisfies this LTL constraint. The accompanying costmap visually differentiates between low-cost areas (depicted in blue) and high-cost zones (shown in yellow), guiding the trajectory to navigate through cost-efficient regions while meeting the mission’s objectives.

Extensive simulation testing confirms the effectiveness of the proposed method in efficiently discovering solutions and minimizing costs. The results demonstrate a significant improvement in performance compared to existing methodologies.

## 2. Related Work

Path planning in robotics and autonomous systems is a diverse field, addressing the complexities of navigating through environments with varying dynamics and specific mission requirements. This section reviews key contributions and emerging trends that directly inform our approach to low-cost, dynamic path planning under Linear Temporal Logic (LTL) specifications.

**Finite deterministic systems**: Research in this area often employs LTL to articulate constraints and objectives, focusing on cost optimization within given mission parameters [27,28]. These works have laid foundational approaches for integrating cost functions into path planning. However, their application to continuous path planning is limited by the reliance on discrete models, which struggle to capture the full dynamics of robotic movement. Our study builds upon these foundational concepts to address the continuous nature of robotic path environments.

**Sampling-based motion planning**: Methods such as Rapidly-exploring Random Trees (RRT) and its variants [29,30,31] have significantly advanced the handling of complex dynamics within path planning. The scalability issues of constructing product automata in these methods [32] highlight a critical bottleneck that we aim to address by integrating more efficient computational techniques and advanced logic formulations in our proposed method.

**Optimization methods**: Techniques transforming LTL specifications into mixed-integer constraints [33,34] have structured the search for optimal paths under strict logical constraints. Our research extends these methodologies by enhancing the computational efficiency and scalability of path planning algorithms, crucial for dealing with complex environments laden with obstacles.

**Multi-layered frameworks**: Multi-layered frameworks [25,35,36,37] represent a significant advancement in path planning by integrating discrete abstraction with sampling-based methods. These frameworks facilitate the identification of feasible trajectories that satisfy complex logic constraints, such as those imposed by Linear Temporal Logic (LTL). Our proposed method builds upon these frameworks to enhance the efficiency and effectiveness of path planning in dynamic environments, where adaptability and compliance with LTL specifications are crucial.

**Learning from demonstration (LfD)** approaches: Learning from demonstration has emerged as a powerful approach to path planning, particularly within the context of autonomous systems [38,39,40,41]. By integrating machine learning techniques with rule-based systems, LfD enables the derivation of robust and interpretable policies that align with predefined safety and operational specifications. Our approach leverages these advancements to refine the path planning process, ensuring that it not only meets technical and safety criteria but also adapts to real-world complexities and learning from actual operational data.

## 3. Preliminaries

This section introduces the basic concepts and system models essential for understanding our path planning approach, focusing on the dynamics and control mechanisms relevant to autonomous systems.

### 3.1. System Model

To define the system model, the following notations are introduced:

- $X\subset R^{n}$: The state space of the system.
- $X_{obs}\subset R^{n}$: The obstacle space.
- $X_{free}=X\setminus X_{obs}$: The free space.
- $U\subset R^{m}$: The control space.
- $W\subset R^{n_{w}}$: The workspace.
- h:X→W: The mapping function.

The dynamic model of the system is described by the following equation:

(1) $${\dot{x}}_{t}=f\left(x_{t},u_{t}\right),$$

where $x_{t}\in X_{free}$ specifically represents a state within the navigable subset of the overall state space X that is free from obstacles. Here, $u_{t}\in U$ denotes the control input, and *f* is a function that is smooth and continuously differentiable, mapping the current state and control input to the rate of change of the state.

The trajectory x of the system, starting from the initial state $x_{0}$ under a given control signal u over the time interval [0,T], is denoted by $x(x_{0},u)$. This trajectory represents a sequence of states as a function of time, capturing the system’s evolution from $x_{0}$ under the influence of u.

To facilitate computational handling and simulation, the trajectory is often discretized. The discretized version of this trajectory, denoted by $x_{\Delta t}\left(x_{0},u\right)$, is defined as

(2) $$x_{\Delta t}\left(x_{0},u\right)={\left\{x\left(x_{0},u,i\Delta t\right)\right\}}_{i=0}^{i_{f}},$$

where Δt represents the time step, chosen from the positive real numbers $R^{+}$, and $i_{f}$ is the final index in the discretization, an integer from N. Each element in this sequence, $x(x_{0},u,i\Delta t)$, corresponds to the state of the system at discrete time intervals iΔt, providing a vector representation of the state at each sampled point.

### 3.2. Linear Temporal Logic (LTL)

Linear temporal logic (LTL) is a logical formalism suited for specifying linear time characteristics [30]. LTL formulas consist of atomic propositions (APs), Boolean operators, and temporal operators. APs are statements that can be either true or false. The LTL operators include ◯ (next), U (until), □ (always), ◊ (eventually), and ⇒ (implication). LTL formulas follow specific grammatical rules [42].

$\Pi =\{\pi_{0},\pi_{1},\ldots ,\pi_{N}\}$ stands for a set of atomic propositions. A trace σ is a sequence of atomic propositions, and LTL semantics are defined over infinite traces. Given a trace σ, the notation σ⊧ϕ signifies that σ satisfies the formula ϕ.

This study concentrates on path planning within a finite time horizon, with a specific focus on syntactically co-safe LTL formulas (sc-LTL) [43]. Sc-LTL represents a subset of Linear Temporal Logic (LTL) formulas, characterized by certain constraints. An LTL formula ϕ is considered co-safe if any infinite trace that satisfies ϕ also has a finite prefix fulfilling ϕ. In this context, all temporal logic formulas discussed and utilized adhere strictly to the sc-LTL criterion. This approach ensures that the path planning solutions developed are not only compliant with the temporal logic requirements but also suitable for finite time horizon scenarios, a crucial aspect for practical applications in robotics and autonomous systems.

#### 3.2.1. Automaton Representation

In the realm of automaton representation, the study engages with nondeterministic finite automata (NFA) construction from a set of atomic propositions Π and syntactically co-safe LTL formulas [44]. For illustration, considering a syntactically co-safe LTL formula such as ϕ=◊(a∧◊(b∧◊(c))), an NFA can be constructed, as depicted in a corresponding figure in the paper. The NFA, while instrumental, can be transformed into a deterministic finite automaton (DFA) for computational efficiency.

A DFA is formalized as $A_{\phi }=\left(Q,\Sigma ,\delta ,q_{init},Q_{acc}\right)$, where the components are defined as follows:

- *Q*: A finite set of states.
- $\Sigma =2^{\Pi }$: A finite alphabet.
- δ:Q×Σ→Q: The transition relation.
- $q_{init}\subseteq Q$: The set of initial states.
- $Q_{acc}\subseteq Q$: The set of accepting states.

**Figure 3:**
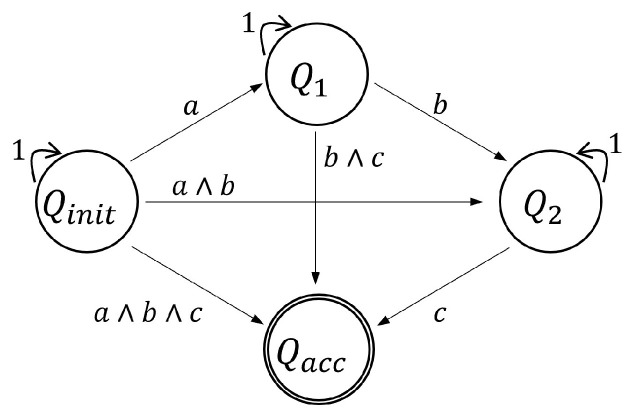
An NFA for the syntactically co-safe LTL formula ϕ=◊(a∧◊(b∧◊(c))). There are four automaton states with input alphabets shown in transition relations.

In this context, an infinite trace σ of a DFA is deemed accepting if its prefix eventually intersects with one of the accepting states in the DFA ($\sigma_{i}\cap Q_{acc}\neq \emptyset$). Therefore, a trajectory $x_{\Delta t}\left(x_{0},u\right)$ is considered to satisfy the LTL formula ϕ if its corresponding trace is accepted by the DFA $A_{\phi }$. Figure 3 illustrates the NFA representing the syntactically co-safe LTL formula ϕ, showcasing its structure consisting of four states and the transition relations for input alphabets.

#### 3.2.2. LTL Semantics over Trajectories

In the workspace W, define regions of interest as $P=P_{1},\ldots ,P_{n}$. Correspondingly, let each atomic proposition $\pi_{j}\in \Pi$ be uniquely associated with a region of interest $P_{j}$. We employ a labeling function $L:W\to 2^{\Pi }$ to map points within the workspace to the atomic propositions that hold true at those locations. For any $\pi_{i}\in \Pi$, the negated proposition $\neg \pi_{i}$ is valid at any workspace point *w* where $\pi_{i}$ is not in the label set L(w). Notably, $\pi_{0}$ holds true universally, except within designated regions of interest and obstructed spaces.

Given a control input u over a discrete time sequence, the trajectory $x_{\Delta t}\left(x_{0},u\right)=x_{0},x_{1},\ldots ,x_{m}$ starting from $x_{0}$ and progressing at intervals Δt, can be represented as a sequence of labels:

(3) $$\begin{array}{cc} \mathrm{trace}(x_{\Delta t}\left(x_{0},u\right))= & L\left(h\left(x_{0}\right)\right),L\left(h\left(x_{1}\right)\right),\ldots ,L\left(h\left(x_{m}\right)\right). \end{array}$$

The corresponding automaton states for a trajectory trace, $trace\left(x_{\Delta t}\left(x_{0},u\right)\right)=\tau_{0},\tau_{1},\ldots ,\tau_{m}$, can be constructed by applying the transition function δ as follows:

(4) $$q_{k}=\left\{\begin{array}{cc} \delta (q_{init},\tau_{0}), & \mathrm{if}\;k=0\\ \delta (q_{k-1},\tau_{k}), & \mathrm{if}\;k\geq 1. \end{array}\right.$$

A trajectory is considered compliant with the LTL formula ϕ, denoted by $x_{\Delta t}\left(x_{0},u\right){⊧}_{\Delta t}\phi$, if it leads to an automaton state within the accepting set $Q_{acc}$.

## 4. Proposed Method

The accumulated cost $J(x_{0},u)$ represents the line integral of the cost function *c* along a trajectory and is defined as follows:

(5) $$J\left(x_{0},u\right)=\frac{1}{T}\int_{0}^{T}c\left(x\left(x_{0},u,t\right)\right)dt,$$

where $c:X\to R^{+}$ is a continuous and bounded cost function, u is a control signal from t=0 to t=T, and $x_{0}$ is the initial state. The mission is defined using a syntactically co-safe LTL formula, where each atomic proposition corresponds to a region of interest.

This study introduces a refined trajectory planning method designed to minimize costs and fulfill Linear Temporal Logic (LTL) mission specifications. This method emphasizes the integration of dynamic models and cost functions, a departure from traditional path planning approaches that often overlook such comprehensive considerations.

By adopting and enhancing the Rapidly exploring Random Tree (RRT) algorithm, this method improves the selection and expansion of search tree vertices. This not only optimizes the efficiency of path planning but also ensures cost-effectiveness. The innovation extends established two-layered path planning strategies, drawing from the strengths of models cited in the literature [25,35,45,46], and enhances them to suit modern, complex scenarios.

A critical advancement of our proposed method is its move away from traditional mesh decomposition techniques, while effective in less complex environments, mesh decomposition can become a bottleneck in more complicated settings. Our method simplifies the planning process with a discrete abstraction approach, eliminating the need for mesh decomposition and consequently providing greater adaptability to varied and challenging environments.

An integral advancement of our method is the innovative application of deep learning within the path planning process. We utilize a Conditional Variational Autoencoder (CVAE) [47], augmented by a SEQ2SEQ structure [48], which together form a powerful data-driven approach to identify cost-efficient path segments. This combination enables a strategic and biased sampling strategy that significantly streamlines the path discovery process. The incorporation of this deep learning mechanism, which is inspired by and expands upon previous research [22,25], adheres to the core tenets of sampling-based planning. It concurrently introduces marked enhancements in efficiency by learning complex patterns and dependencies in path cost data, facilitating the generation of solutions that are both cost-effective and aligned with stringent mission specifications.

The advantages of the proposed method include the following:

1. **Enhanced efficiency:** This method’s focus on optimizing vertex selection and extension in the search tree leads to more efficient trajectory planning.
2. **Flexibility in complex environments:** By eliminating the need for mesh decomposition, this method is more adaptable to intricate and high-dimensional environments.
3. **Improved cost-effectiveness:** The deep learning component allows for a more targeted search for low-cost paths, aligning closely with mission requirements.
4. **Scalability:** The use of a deep learning framework offers scalability and adaptability to a range of different path planning scenarios and constraints.

Overall, this proposed method marks a substantial progression in the field of path planning. It offers a robust, efficient, and adaptable framework for navigating complex environments, meeting detailed mission specifications, and optimizing path costs, thereby setting a new benchmark for future trajectory planning endeavors.

### 4.1. Minimal Discrete Abstraction

Discrete abstraction serves to construct a simplified representation of the problem, focusing on environmental geometry, such as obstacles. Its primary function is to facilitate the search tree’s compliance with the specified LTL. The abstraction utilizes a straightforward graph, $D=(R_{d},E_{d})$, which delineates the geometric structure of both regions of interest *P* and non-obstacle zones that are also not regions of interest, denoted *W*. Vertices are defined as $R_{d}=W\cup P$, while edges are represented by $E_{d}=\left\{\left(r_{i},r_{j}\right):r_{i},r_{j}\in R_{d}\;\mathrm{and}\;\mathrm{are}\;\mathrm{adjacent}\right\}$.

The proposed method takes the cross product of the graph D with the DFA of the syntactically co-safe LTL formula ϕ, resulting in a *minimal discrete abstraction*. This abstraction differs only marginally in size from the DFA, primarily due to the deliberate avoidance of mesh decomposition. Following the establishment of this abstraction, discrete planning is employed to generate discrete plans. These plans comprise a sequence of discrete states, each of which is represented as a pair 〈q,r〉, with $q\in A_{\phi }.Q$ and $r\in R_{d}$. This approach provides a streamlined and efficient means to navigate the planning space while adhering to the designated mission criteria.

### 4.2. Search Tree

The central data structure of the proposed algorithm is a search tree, denoted as T, which comprises vertices T.V and edges T.E. The root of this tree, $v_{init}$, represents the initial state of the system. Each vertex within the tree contains crucial planning information, encapsulating various aspects of the robot’s status. These include the robot’s state (expressed as v.x), the discrete region it occupies (v.r), the current state of the automaton (v.q), and the accumulated trajectory cost from the initial state $v_{init}$ to the current vertex (v.c).

Edges within the tree provide insights into the control inputs and time intervals required to transition between two vertices, $v_{i}$ and $v_{j}$. The algorithm manages vertices with identical high-level states by grouping them into sets based on their automaton state and discrete region. This grouping is mathematically represented as follows:

(6) $$\Gamma_{\langle q^{\prime },r^{\prime }\rangle }=\left\{v\in T.V\;|\;v.q=q^{\prime },v.r=r^{\prime }\right\}.$$

Such organization within the search tree allows for an efficient and structured approach to planning, ensuring that the algorithm systematically navigates through the planning space while conforming to the specified LTL mission specifications and dynamic constraints.

### 4.3. Algorithm

The algorithm is described in Algorithm 1. It takes the following as inputs:

- DFA of a syntactically co-safe LTL formula, denoted as $A_{\phi }$.
- Workspace information, $D=(R_{d},E_{d})$.
- Robot’s initial state, $x_{init}$.
- Initial automaton state, $q_{init}$.

The search tree, symbolized as T, begins with the initialization of $x_{init}$ and $q_{init}$. This approach stands out from traditional two-layered path planning methods by determining discrete plans prior to the main iteration rather than during it. The advantage of this early search is derived from employing a minimal discrete abstraction.

Discrete plans, represented as $\Sigma_{P}$ and compliant with the LTL, are generated from the initial discrete state. These plans are obtained using Dijkstra’s algorithm on the product space $A_{\phi }.Q\times D$, with edge costs uniformly set to 1. This uniform cost assignment ensures that while the discrete plans steer the search tree in accordance with the LTL specifications, they do not inherently prioritize low-cost trajectories.

As the iterative process unfolds, the search tree T continues to expand. Each iteration involves the algorithm selecting a starting discrete state, $\langle q_{s},r_{s}\rangle$, based on the current configuration of the search tree. Subsequently, a target discrete state is identified from the set of discrete plans $\Sigma_{P}$ and the selected starting state. The expansion of the tree T then proceeds towards the targeted discrete region $r_{t}$. Upon the completion of these iterations, the algorithm identifies the optimal trajectory that fulfills the LTL formula ϕ as the final solution. Further sections of the paper delve into more detailed descriptions of each of these steps, providing a comprehensive understanding of the process and its underlying mechanisms.
**Algorithm 1** Proposed method1:$T.V\leftarrow \{x_{init},q_{init}\}$2:T.E←∅3:$\Sigma_{P}\leftarrow$ DiscretePlanning($A_{\phi },D,\langle q_{init},r_{init}\rangle$)4:**while** time()<tmax **do**5:    $\langle q_{s},r_{s}\rangle \leftarrow$ GetInitialState(T)6:    $\langle q_{t},r_{t}\rangle \leftarrow$ GetTargetState($T,\Sigma_{P},\langle q_{s},r_{s}\rangle$)7:    T← ExtendTree($A_{\phi },T,\Gamma_{\langle q_{s},r_{s}\rangle },r_{t}$)8:**end while**9:**if** $\exists v_{i}\in T.V$ s.t. $v_{i}.q\in A_{\phi }.Q_{acc}$ **then**10:    return Ξ← SelectMinTraj(T)11:**else**12:    return null13:**end if**

#### 4.3.1. Selection of an Initial State

The function *GetInitialState* chooses an initial discrete state $\langle q_{s},r_{s}\rangle$ for expansion. This choice is influenced by the exploration data from the search tree, assigning weights to each discrete state. The weight of a discrete state 〈q,r〉 is formulated as follows:

(7) $$w\left(q,r\right)\propto \frac{\mathrm{Area}(r)}{\left(1+\right|\Gamma_{\langle q,r\rangle }{\left|\right)}^{\alpha }},$$

where Area(*r*) is the area of region *r*, and $|\Gamma_{\langle q,r\rangle }|$ signifies the number of vertices linked to 〈q,r〉. The positive real number α adjusts the effect of the vertex count. Building upon prior research [25,35,45,46], this approach has been modified for the main problem of this paper.

States are chosen probabilistically from the set of discrete states that includes the search tree’s vertices. The selection probability is directly proportional to w(q,r), favoring larger discrete regions and states less explored previously.

#### 4.3.2. Selection of a Target State

The *GetTargetState* function determines the target discrete state for pursuit, aiming to steer the search tree according to the given LTL specification or to inspect other regions. This study employs two methods for target state selection:

1. **Transitioning to the next automaton state:** Initially, a discrete plan $\sigma_{P}$ from the plan set $\Sigma_{P}$ that includes the starting state $\langle q_{s},r_{s}\rangle$ is selected. This plan helps identify the target state $\langle q_{t},r_{t}\rangle$ to proceed to the next automaton state. If $\sigma_{P}$ is defined as
  (8) $$\sigma_{P}=\langle q_{init},r_{init}\rangle ,\langle q_{1},r_{1}\rangle ,\cdots \langle q_{M},r_{M}\rangle ,$$
  and the *i*-th element in $\sigma_{P}$ is the starting state $\langle q_{i},r_{i}\rangle =\langle q_{s},r_{s}\rangle$, then $\sigma_{P,i}$ is the subset from the *i*-th index:
  (9) $$\sigma_{P,i}=\langle q_{i+1},r_{i+1}\rangle ,\cdots \langle q_{M},r_{M}\rangle .$$
  From this subset $\sigma_{P,i}$, we select the target state $\langle q_{t},r_{t}\rangle$. The criteria for this selection are as follows: $r_{t}$ should belong to the regions of interest (i.e., $r_{t}\in \left\{P_{1},\ldots ,P_{n}\right\}$), and $q_{t}$ should be the first automaton state that is different from $q_{s}$.
2. **Random state selection:** The target state is chosen randomly from the set
  (10) $$\{\langle q,r\rangle \;|\;q=q_{s},r\in R_{d}\},$$
  enabling the tree to explore within the automaton state $q_{s}$.

The first strategy prioritizes alignment with the LTL specification, whereas the second examines all possible states. A random choice determines the employed strategy: the first has a chance of $p_{D}\in \left[0,1\right)$, while the second has a probability of $1-p_{D}$.

#### 4.3.3. Search Tree Expansion

The tree expansion algorithm, as outlined in Algorithm 2, builds upon the foundations established in previous studies [25]. The novel contribution of this work lies in the integration of a deep learning network. This network is specifically tailored to identify control sequences that effectively direct the path towards the target point, while simultaneously considering the cost configurations involved. This integration of deep learning into the tree expansion process represents a significant advancement, enhancing the algorithm’s ability to navigate complex environments and achieve cost-effective trajectories in alignment with the predefined LTL mission criteria.

The process begins by uniformly selecting a target point $p_{t}$ from $r_{t}$ (line 1). From the tree partition $\Gamma_{s}$, the initial vertex $v_{s}$ is either randomly chosen from the vertices of T in $\Gamma_{s}$ with a probability $p_{T}\in \left[0,1\right)$ (line 2) or the vertex nearest to $p_{t}$ is designated as $v_{s}$ with probability $1-p_{T}$. A path is then sought, initiating from $v_{s}$ and moving towards $p_{t}$.
**Algorithm 2** ExtendTree($A_{\phi },T,\Gamma_{s},r_{t}$)1:$p_{t}\leftarrow$ SelectTargetPoint($r_{t}$)2:$v_{s}\leftarrow$ SelectInitialVertex($T,\Gamma_{s},p_{t}$)3:**if** $∥v_{s}.x-p_{t}∥$$>\delta_{d}$ **then**4:    $\zeta_{seg}\leftarrow \mathrm{DL}\_\mathrm{Extend}\left(v_{s}.x,p_{t}\right)$5:**else**6:    $\zeta_{seg}\leftarrow \mathrm{Steer}\left(v_{s}.x,p_{t}\right)$7:**end if**8:**if** CollisionFree($\zeta_{seg}$) **then**9:    T← UpdateTree($A_{\phi },T,v_{s},\zeta_{seg}$)10:**end if**11:return T

If the distance between $v_{s}.x$ and $p_{t}$ surpasses a threshold $\delta_{d}$, the deep-learning-based *DL_Extend* function is employed to discover a cost-efficient path from $v_{s}.x$ to $p_{t}$ (line 4). Further details of *DL_Extend* are elucidated in the subsequent section. Alternatively, the familiar *Steer* function from sampling-based motion planning algorithms [26] is utilized. Once the generated path segment $\zeta_{seg}$, initiating from $v_{s}.x$ and targeting $p_{t}$, is verified as valid, it is incorporated into the search tree T (line 9).

Algorithm 3 presents the *UpdateTree* function, which plays a crucial role in the tree expansion algorithm. In this context, $Q_{feas}$ represents the set of automaton states that have the capability to reach accepting automaton states. A key aspect of this function is the integration of the rewiring procedure from the ${\mathrm{RRT}}^{*}$ algorithm. This procedure is applied to refine the cost associated with a vertex in T after evaluating neighboring vertices. Through this iterative process of refinement, the algorithm effectively converges towards a solution that is near-optimal. This approach enhances the algorithm’s efficiency and accuracy in determining cost-effective paths that align with the specified LTL mission requirements.

Each node in the path segment $\zeta_{seg}$ undergoes the *UpdateVertex* operation, refreshing information for the newly appended vertex (line 3): the robot’s state $x_{i}$, the decomposed region $\Upsilon_{d}\left(h\left(x_{i}\right)\right)$, the automaton state $A_{\phi }.\delta \left(v_{parent}.q,L\left(h\left(x_{i}\right)\right)\right)$, and the trajectory cost from the initiating vertex to the current one $v_{parent}.c+\cos t\left(\mathrm{edge}\left(v_{parent},v_{i}\right)\right)$. The iteration is terminated if the automaton state of the new vertex is not part of the feasible automaton states $Q_{feas}$ (line 4).

The function Near(x) yields a set of vertices, $V_{near}$, in T that reside within a radius $\rho =\gamma {\left(\log n/n\right)}^{1/d}$ centered at h(x). *n* is the vertex count in T and *d* denotes the state dimension. A sufficient γ value ensures the detection of a practicable number of rewiring candidates.

The function *FeasibilityCheck* within the algorithm plays a pivotal role in verifying the legitimacy of connecting two vertices in the search tree. To establish a legitimate connection between any two vertices, the algorithm requires the fulfillment of two key conditions: first, the edge connecting the vertices must be free of any collisions, and second, there must be a match in the automaton state between the computed vertex and the existing vertex in the tree.

This rigorous process of verification and comparison incrementally refines the solution, ensuring its alignment with the provided Linear Temporal Logic (LTL) specification. This alignment is critical for guiding the algorithm towards an optimal solution. Specifically, lines 11–16 of the algorithm are dedicated to determining the minimum cost path for every newly added vertex. Following this, the rewiring procedure, which is an integral part of the ${\mathrm{RRT}}^{*}$ algorithm, is executed between lines 19–26. The implementation of these steps within the *FeasibilityCheck* function is crucial for maintaining the algorithm’s integrity and effectiveness in finding a feasible and cost-efficient path that adheres to the specified mission criteria.
**Algorithm 3** UpdateTree($A_{\phi },T,v_{s},\zeta_{seg}$)1:$v_{parent}=v_{s}$2:**for** $x_{i}\in \zeta_{seg}:\left\{x_{i}\in X\right\}$ **do**3:      $v_{i}\leftarrow \mathrm{UpdateVertex}\left(v_{parent},x_{i},A_{\phi }\right)$4:      **if** $v_{i}.q\notin Q_{feas}$ **then**5:        break;6:      **end if**7:      $T.V\leftarrow T.V\cup \{v_{i}\}$8:      $v_{min}\leftarrow v_{parent}$9:      $c_{min}\leftarrow v_{i}.c+\cos t\left(\mathrm{edge}\left(v_{min},v_{i}\right)\right)$10:    $V_{near}\leftarrow \mathrm{Near}\left(v_{i}.x\right)$11:    **for each** $v_{near}\in V_{near}\setminus \left\{v_{parent}\right\}$ **do**12:        $c_{near}\leftarrow v_{near}.c+\cos t\left(\mathrm{edge}\left(v_{near},v_{i}\right)\right)$13:        **if** $\mathrm{FeasibilityCheck}\left(A_{\phi },v_{near},v_{i}\right)\wedge c_{near}<c_{min}$ **then**14:           $v_{min}\leftarrow v_{near},c_{min}\leftarrow c_{near}$15:        **end if**16:    **end for**17:    $T.E\leftarrow T.E\cup \{\left(v_{min},v_{i}\right)\}$18:    $V_{near}^{\prime }\leftarrow \mathrm{Near}\left(v_{i}.x\right)$19:    **for each** $v_{near}\in V_{near}^{\prime }$ **do**20:        $c_{new}\leftarrow v_{i}.c+\cos t\left(\mathrm{edge}\left(v_{i},v_{near}\right)\right)$21:        **if** $\mathrm{FeasibilityCheck}\left(A_{\phi },v_{i},v_{near}\right)\wedge c_{new}<v_{near}.c$ **then**22:           $v_{near}.c\leftarrow c_{new}$23:           $T.E\leftarrow T.E\setminus \{\mathrm{edge}\left(\mathrm{Parent}\left(v_{near}\right),v_{near}\right)\}$24:           $T.E\leftarrow T.E\cup \{\mathrm{edge}\left(v_{i},v_{near}\right)\}$25:        **end if**26:    **end for**27:    $v_{parent}=v_{i}$28:**end for**

#### 4.3.4. Deep-Learning-Based Extension

The *DL_Extend* function is an integral component of the algorithm, specifically designed to identify low-cost path segments connecting two points. In the context of Algorithm 2 (line 4), $\mathrm{DL}\_\mathrm{extend}(v_{s}.x,p_{t})$ is employed to generate a path segment starting from point $v_{s}.x$ and targeting the endpoint $p_{t}$. The primary aim of this function is to expedite the identification of low-cost trajectory options within the planning process. To achieve this, the function strategically guides the sampling towards areas with high potential for low-cost paths, utilizing a learned sample distribution to ascertain the most efficient route.

**Figure 4:**
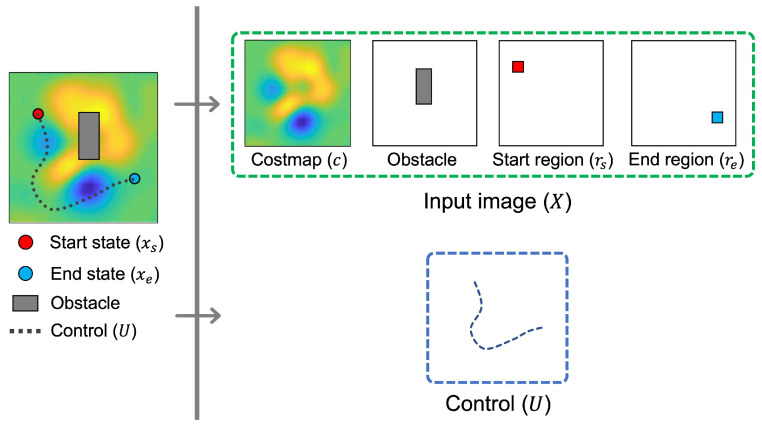
Input and output data configuration for the proposed deep learning network, involving the optimal path connecting the starting state and end state.

Prior to delving into the specifics of the deep learning network, it is essential to define the structure of the input and output data used by the network, as illustrated in Figure 4.

The process begins with the derivation of an optimal trajectory and corresponding control inputs that connect an initial state $x_{s}$ to a final state $x_{e}$, taking into account both the costmap *c* and obstacles obs. An input image, denoted as *X*, is then constructed based on this information. This composite image is formed by concatenating four distinct elements: the costmap *c*, the obstacle map obs, and the starting ($x_{s}$) and ending ($x_{e}$) states. As a result, a four-channel image is produced, in which the starting and ending states are depicted as regions $r_{s}$ and $r_{e}$, each positioned within its respective channel. This approach ensures a comprehensive representation of the planning environment, facilitating the deep learning network’s ability to accurately predict low-cost path segments.

In the training phase of the proposed deep learning network, the generated input image *X* is utilized as the input data, whereas the control *U* is considered the output data. It is crucial to emphasize that the strategy implemented for curating the training dataset is meticulously aligned with this defined input–output structure. This alignment ensures that the deep learning model is effectively trained on data that are representative of the actual scenarios it will encounter, thereby enhancing its ability to accurately predict control sequences for low-cost path segments in the context of the path planning algorithm.

The architecture presented in this paper is based on the use of a Conditional Variational Autoencoder (CVAE) [47], which is an enhanced form of the standard variational autoencoder. The CVAE is particularly adept at conditional data generation, enabling the sampling of data from the model’s latent space. In the context of motion planning, these conditioning variables include input features relevant to the current planning scenario, with the output representing the trajectory or path segment.

The application of CVAE in sampling-based path planning algorithms has been previously investigated [25]. The method developed in this study further refines the use of the deep learning network, placing a special emphasis on the generation of control sequences. This concept of generating control sequences using deep learning has been previously explored in motion forecasting research [49,50]. The refinement aims to enhance the network’s capability to accurately predict control inputs for path segments, thereby improving the efficiency and effectiveness of the path planning process.

**Figure 5:**
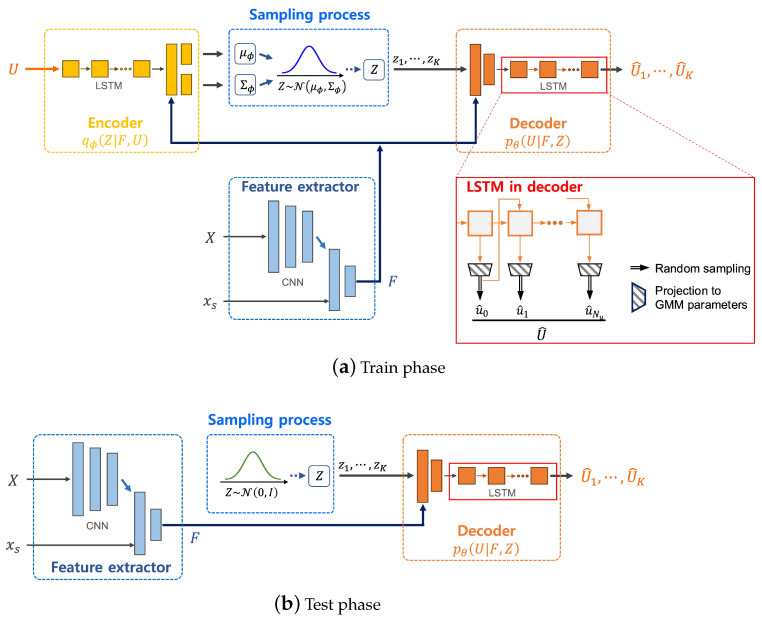
Training and testing phases of the proposed architecture. Predicted values employ the hat notation for representation. Components like the costmap (*c*), obstacles (obs), start state ($x_{s}$), and end state ($x_{e}$) are merged into feature *F* via the *Feature extractor* network. The output control sequence samples are represented by ${\hat{U}}_{1},\cdots ,{\hat{U}}_{K}$ and are transmuted into trajectories through the dynamic model (1). In the testing phase, the decoder operates online to convert conditioned latent samples $z_{1},\cdots ,z_{K}$ into the control distribution.

Figure 5 presents a detailed overview of the network architecture proposed in the study. The process begins with the extraction of the feature *F* from the input image *X* and the initial state $x_{s}$ using a *Feature extractor* network. This extracted feature *F* serves as the conditional variable in the Conditional Variational Autoencoder (CVAE) framework.

Consistent with the CVAE methodology, the architecture defines three parameterized densities: the recognition model $q_{\phi }\left(Z|F,U\right)$, the (conditional) prior model $p_{\theta }\left(Z|F\right)$, and the generation model $p_{\theta }\left(U|F,Z\right)$, where θ and ϕ are the parameter vectors. For the sake of clarity, the notations θ and ϕ are specifically used in this context.

Both the encoder and decoder segments of the network incorporate Long Short-Term Memory units (LSTMs) [51], a choice aimed at capturing the temporal dynamics effectively. In the decoder model, particularly, the control outputs are structured as a Gaussian Mixture Model (GMM). This configuration allows for a more nuanced and accurate representation of the real data distribution. The input for each LSTM cell in the sequence is sampled from this GMM, facilitating the generation of control sequences that are reflective of the underlying data characteristics. This approach significantly enhances the network’s ability to predict control outputs that are conducive to efficient and effective path planning.

In our study, we formalized the three parameterized density models in the subsequent manner:

- The posterior density of the latent variables, given features *F* and control sequence *U*, is modeled as a Gaussian distribution:
  $$q_{\phi }\left(Z|F,U\right)=N\left(\mu_{\phi }\left(F,U\right),\Sigma_{\phi }\left(F,U\right)\right),$$
  where functions $\mu_{\phi }\left(F,U\right)$ and $\Sigma_{\phi }\left(F,U\right)$ are outputs from an encoder neural network.
- The prior density of the latent variables, given only the features *F*, is set as a standard Gaussian:
  $$p_{\theta }\left(Z|F\right)=N\left(0,I\right).$$
- The conditional probability of the control sequence, given the features *F* and latent variables *Z*, is expressed as a product of conditional probabilities for each control output:
  $$p_{\theta }\left(U|F,Z\right)=\prod_{i=0}^{N_{u}}p_{\theta }\left(u_{i}|F,Z,u_{0:i-1}\right),$$
  depicting the sequential nature of generating the control sequence $U=u_{0},\cdots ,u_{N_{u}}$, with $N_{u}$ denoting the length of the sequence.

The log-likelihood of observing the control sequence *U* given the features *F* can be broken down into two components, described by the Evidence Lower Bound (ELBO):

(11) $$\begin{array}{cc} \log p_{\theta }\left(U|F\right)-D_{KL}\left(q_{\phi }\left(Z|F,U\right)\left|\right|p_{\theta }\left(Z|F,U\right)\right)= & E_{q_{\phi }\left(Z|F,U\right)}\left[\log p_{\theta }\left(U|F,Z\right)\right]\\ \; & -D_{KL}\left(q_{\phi }\left(Z|F,U\right)\left|\right|p_{\theta }\left(Z|U\right)\right). \end{array}$$

Here, $D_{KL}$ denotes the Kullback–Leibler divergence, a measure of how one probability distribution diverges from a second, reference probability distribution.

Subsequently, the ELBO elucidated above is utilized to derive our loss function:

(12) $$-\sum_{k=1}^{K}\sum_{i=0}^{N_{u}}\log\left(p_{\theta }\left(u_{i}^{\left(k\right)}|F,z_{k},u_{0:i-1}^{\left(k\right)}\right)\right)+\lambda \cdot D_{KL}\left(N\left(\mu_{\phi }\left(F,U\right),\Sigma_{\phi }\left(F,U\right)\right)\left|\right|N\left(0,I\right)\right).$$

where λ is a hyperparameter that balances the reconstruction accuracy and the divergence penalty.

The loss function employed in the network architecture accounts for control sequences derived from *K* latent samples within the reconstruction loss term. The value of λ is set to 1. The optimization of this model involves minimizing the loss function, focusing on the parameters θ and ϕ. This optimization process is critical for refining the network’s ability to accurately generate control sequences, ensuring that the resulting path planning aligns effectively with the specified objectives and constraints.

In the testing phase, the decoder is employed online to project conditioned latent samples $z_{1},\cdots ,z_{K}$ into control sequences ${\hat{U}}_{1},\cdots ,{\hat{U}}_{K}$. Control sequences that extend beyond the reach of the end state $x_{e}$ are disregarded. Path segments are then reconstructed from these generated control values. The process involves selecting the path segment with the minimum cost from these reconstructed segments, which is subsequently used as the output of the *DL_Extend* function. This approach ensures that the most cost-effective trajectory is chosen, aligning with the overall objective of optimizing path planning based on the deep learning-augmented algorithm.

#### 4.3.5. Solution Determination Process

Upon completion of the iterative process, the algorithm identifies a valid solution if it locates a vertex corresponding to one of the accepting states, denoted by $A\phi .Q_{acc}$. In instances where such a vertex remains unidentified, the algorithm outputs an empty set, signifying the absence of a viable solution within the designated computational time frame.

If a solution is pinpointed, the algorithm proceeds to select the most cost-effective vertex among those associated with the accepting states of the automaton. The path to the solution, represented as Ξ, is reconstructed by tracing back from this optimal vertex to the tree’s root (as outlined in Algorithm 1, line 10).

## 5. Experimental Results

The simulation showcases outcomes utilizing the Dubins car model, governed by the dynamics

(13) $$\dot{x}=v\cos\left(\theta \right),\;\dot{y}=v\sin\left(\theta \right),\;\dot{\theta }=\omega ,$$

with the car’s location represented by (x,y), its orientation by θ, and *v* and ω indicating its linear and angular velocities, respectively. For this simulation, we chose planning parameters $p_{D}=0.7$ and $p_{T}=0.5$.

For the training of the *DL_extend* function in Algorithm 2, the dataset was generated using costmaps created through Gaussian process regression, which incorporated random obstacles. The control values connecting two states were computed using a motion planning method detailed by Kobilarov et al. [52]. This method has been foundational in shaping our approach, particularly in generating realistic control values that reflect varying environmental conditions.

In our study, we constructed a comprehensive training dataset comprising 1000 pairs of positions and control inputs from each of the 500 generated costmaps, totaling 500,000 samples. We allocated 95% of these samples, or 475,000 pairs, for training our deep learning model, while the remaining 5%, or 25,000 samples, were used as validation data to monitor and refine the model’s performance during the training process.

This dataset is crucial in enhancing the robustness and accuracy of the *DL_extend* function, as it enables the model to reflect diverse and realistic navigation scenarios effectively.

Our model’s architecture and the corresponding training parameters are detailed as follows:

- **Feature extractor:** The initial stage of our model consists of four convolutional layers followed by two fully connected layers. These layers have dimensions of 256 and 128 for the hidden layers, respectively. This stage is crucial for extracting and compressing spatial features from the input data, which significantly influences the subsequent stages of trajectory prediction. The output dimension of the feature extractor is configured to 64.
- **Dimension of latent variable:** The dimension of the latent space within our model is set to 32. This specification balances the complexity of the model with computational efficiency, allowing for effective feature representation and subsequent trajectory generation.
- **LSTM encoder–decoder:** At the core of our model is an LSTM-based encoder–decoder structure, where both the encoder and decoder are equipped with 64-dimensional LSTM units. This setup is designed to handle the sequential nature of the control signals *u*, structured as three-dimensional arrays with dimensions (Batch x Timestep x Control_dim). It captures temporal dependencies essential for accurate predictions of future states based on past and present control inputs.
- **GMM projection module:** After decoding, the output passes through a GMM projection module, comprising two fully connected layers with dimensions of 256 each. This module converts the LSTM outputs into a probabilistic framework, depicting potential trajectories as a mixture of Gaussian distributions. This feature is particularly beneficial in dynamic environments where inherent uncertainties must be managed.
- **Training regime:** The network was comprehensively trained for 300 epochs, with a batch size of 32 to balance between computational efficiency and effective learning. The chosen learning rate of $1\times 10^{-3}$ and a weight decay of $1\times 10^{-5}$ helped in fine-tuning the model parameters to minimize overfitting while optimizing performance.

The experimental setup was designed to evaluate the model’s capability to generate feasible trajectories under varying conditions. The results showcased the model’s robustness, with significant improvements in trajectory optimization demonstrated across a range of test scenarios. Figure 5 provides an overview of the network architecture, and detailed results from the training process are discussed, illustrating the effectiveness of the model in achieving low-cost, feasible trajectories within specified LTL constraints.

The loss function used for training our model, crucial for evaluating the efficacy of the trajectory predictions, is defined in Equation (12) of our manuscript. This equation is integral to understanding how the model minimizes errors and optimizes trajectory outputs during training.

The deep learning network and its training were implemented using PyTorch, and the algorithm was developed in Python. The graphical representations in this paper were generated using MATLAB’s image tool.

Three simulation experiments were conducted to demonstrate the proposed algorithm’s versatility and effectiveness. The initial experiment utilized a generated costmap to provide a controlled environment for baseline validation. The subsequent experiment employed an actual traffic accident density map from the city of Helsinki to illustrate the algorithm’s practical applicability in real-world settings. The final experiment was designed to assess the algorithm’s convergence toward the optimal solution, verifying its ability to achieve asymptotic optimality. Together, these simulations underscore the adaptability of the algorithm to a variety of scenarios.

### 5.1. The Generated Costmap

Three toy scenarios were explored, each with distinct costmaps, encompassing planning environments, regions of interest, and obstacles. These scenarios were tested against the following three Linear Temporal Logic (LTL) formulas:

$$\begin{array}{c} \phi_{toy1}=◊\left(a\wedge ◊\left(b\right)\right)\\ \phi_{toy2}=◊\left(a\wedge ◊\left(b\wedge \left(◊c\right)\right)\right)\\ \phi_{toy3}=◊\left(a\right)\wedge ◊\left(b\right)\wedge ◊\left(c\right) \end{array}$$

While $\phi_{toy1}$ and $\phi_{toy2}$ define sequential missions, $\phi_{toy3}$ outlines a coverage mission.

**Figure 6:**
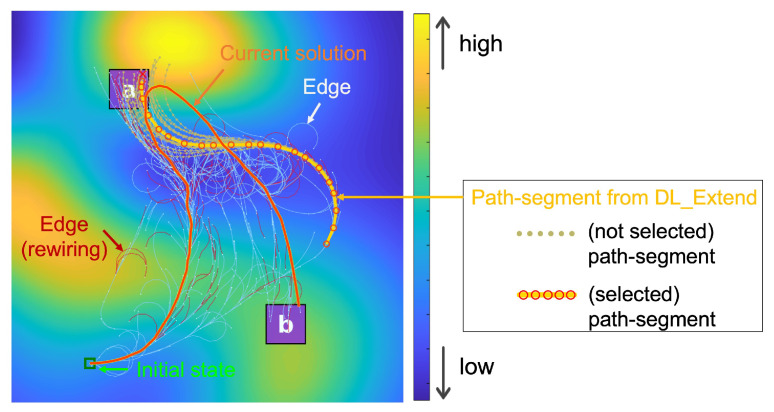
A snapshot of the tree extension process. Within the learning-enhanced extension, various path segments traverse the low-cost region, and the minimum-cost path segment chosen to extend the search tree.

A visual snapshot of the tree extension process at a specific iteration is illustrated in Figure 6. This shows that path segments generated by the deep learning-enhanced extension predominantly traverse low-cost regions. Additionally, edges of the search tree modified by the rewiring process are displayed.

**Figure 7:**
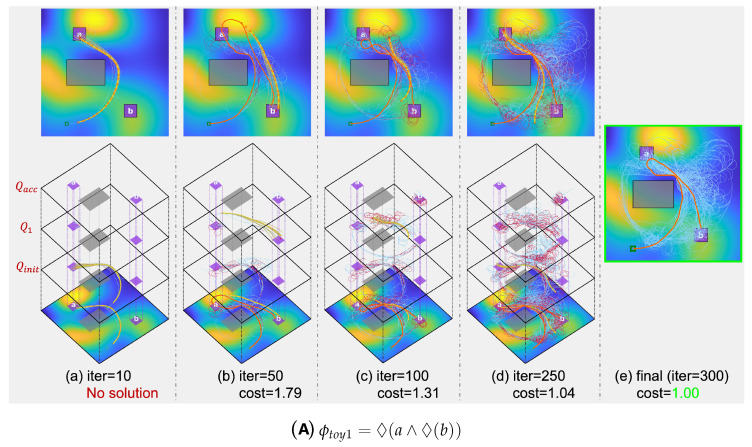

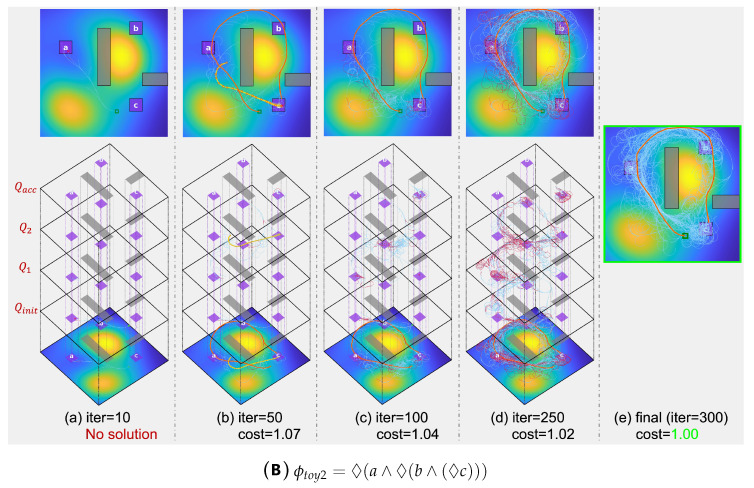
Visualization of the tree extension process for LTL formulas $\phi_{toy1}$ and $\phi_{toy2}$ using the proposed algorithm. In the cost map, areas in blue signify low cost, while those in yellow indicate high cost. The extent of tree extension for distinct iteration steps is illustrated in both 2D and 3D. For the 3D visualizations, distinct layers exist, with each layer signifying the search tree associated with a specific automaton state. Annotations beneath each depiction provide insights into the trajectory cost (scaled against the normalized final solution cost) and its compliance with the respective LTL formula.

**Figure 8:**
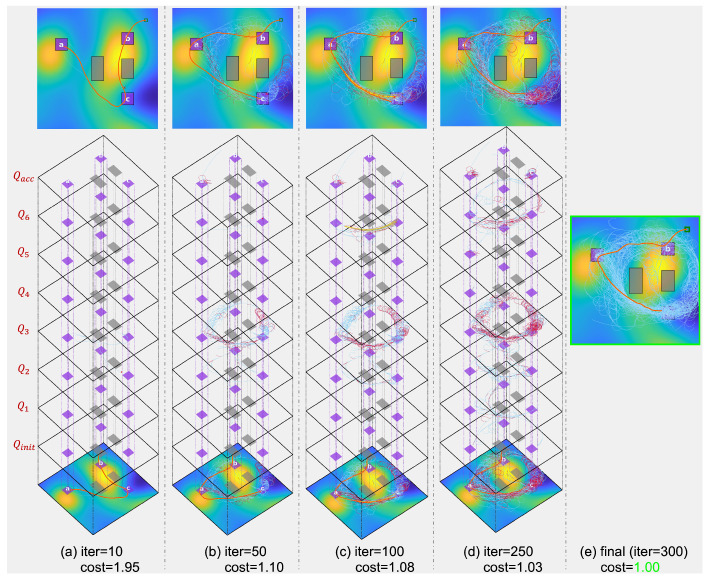
Visualization of the tree extension process using the proposed algorithm for LTL formula $\phi_{toy3}=◊\left(a\right)\wedge ◊\left(b\right)\wedge ◊\left(c\right)$. Refer to Figure 7 for detailed annotations and explanations.

The proposed algorithm’s tree extension processes for various toy scenarios are detailed in Figure 7 and Figure 8. Over 300 iterations, the algorithm’s progression is depicted through 2D and 3D images, showcasing the expansion of the search tree at each stage (column 1∼4). For insights on the 2D images, refer to Figure 6. The final column presents the 2D representation of the solution after 300 iterations, highlighting the solution trajectory (in orange) alongside the search tree (in sky blue).

**Figure 9:**
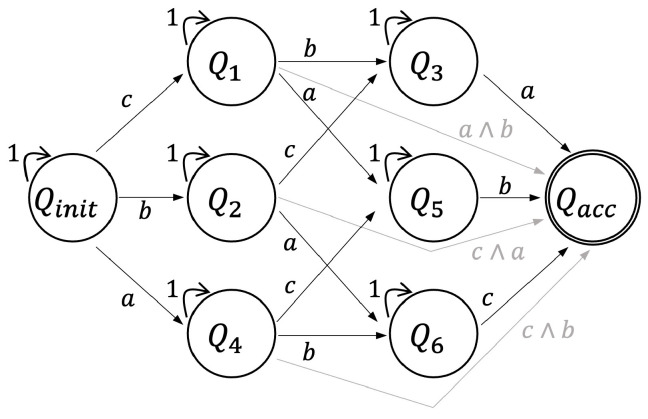
An NFA representing the LTL formula $\phi_{toy3}=◊\left(a\right)\wedge ◊\left(b\right)\wedge ◊\left(c\right)$ with 8 automaton states.

In the 3D images, individual layers correspond to specific automaton states. For instance, Figure 7B displays layers linked to automaton states $Q_{init}$, $Q_{1}$, $Q_{2}$, and $Q_{acc}$. Separately, the NFA for $\phi_{toy3}$, illustrated in Figure 9, includes 8 automaton states, a result of its coverage mission specification. Concurrent tree extensions across multiple automaton states can be observed in Figure 8. By comparing intermediary solution costs to the final solution’s cost (normalized to 1) at the 300th iteration, the algorithm’s continuous refinement is evident. This enhancement is facilitated by a strategic rewiring process that frequently adjusts the edges of the search tree.

Comparative experiments, detailed in Table 1, benchmark the novel proposed approach against a variety of sampling-based algorithms in the context of path planning. In the table, “Proposed” denotes the new method introduced in this study, while “Proposed (wo/r)” represents the same method but excludes the rewiring processes. This approach is compared with two cost-aware path planning algorithms specifically designed for LTL missions: LBPP-LTL [25] and CAPP-LTL [46]. Additionally, the study includes comparisons with two LTL-constrained, sampling-based path planning algorithms: Bahita [35] and McMahon [37]. These algorithms are iterative, operating until reaching a predefined time limit, at which point the solution with the lowest cost is selected.

The results for three distinct scenarios, labeled $\phi_{toy1}$, $\phi_{toy2}$, and $\phi_{toy3}$, are compiled in Table 1. Each algorithm was subjected to 100 independent trials, with each trial introducing a different start state. The operating time for all algorithms was fixed at 420 s, and interim assessments were conducted at 60 second intervals after the initial 120 s. For a clear comparative analysis, a relative average cost metric was employed. The final average cost of the “Proposed” approach (at time = 420 s) served as the baseline (1), and the performance of other algorithms was measured relative to this benchmark.

The analysis reveals that the “Proposed” algorithm consistently identifies more cost-efficient paths, achieving the best average cost after 420 s. A notable observation is the importance of the rewiring function, as evidenced by the comparison with “Proposed (wo/r)”. Although the initial performance of the rewiring process may seem modest, its efficiency becomes more pronounced over time. The study also highlights that the proposed method outperforms other cost-focused path planning algorithms like LBPP-LTL and CAPP-LTL. The divergence of CAPP-LTL from the proposed method is attributed to its reliance on a more time-intensive cross-entropy optimization, as opposed to the deep learning-based extension used in the proposed method. While both the proposed method and LBPP-LTL incorporate a learning-based extension during the tree extension phase, LBPP-LTL differs by requiring discrete planning in each iteration and not utilizing the specialized deep learning network for control sequence generation that is a feature of the proposed method. Conversely, non-cost-centric methods like Bahita and McMahon did not demonstrate significant reductions in the cost value as the time elapsed.

**Figure 10:**
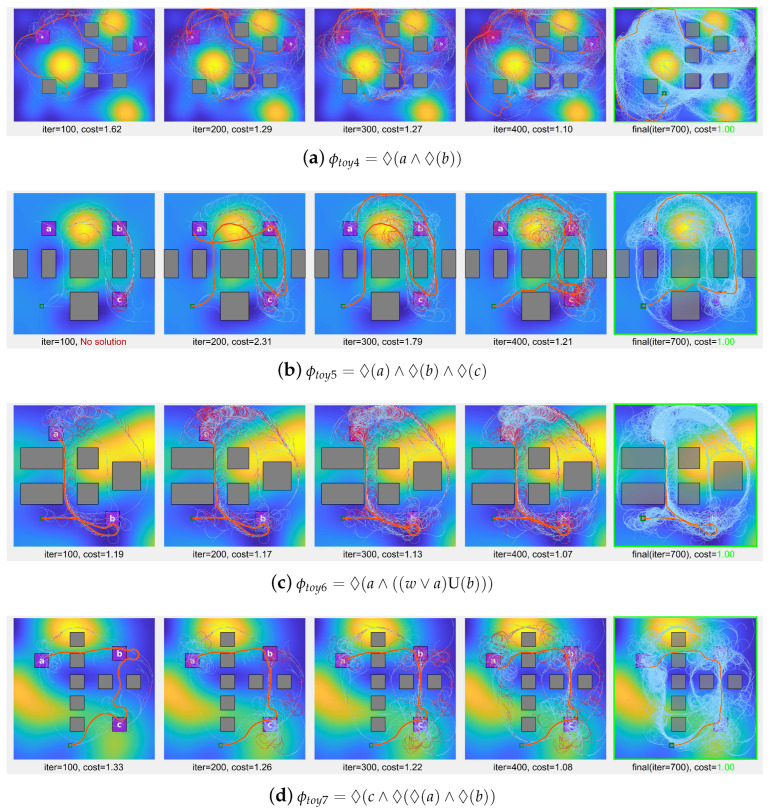
Solution paths and search tree progression demonstrated by the proposed algorithm in scenarios $\phi_{toy4}$ to $\phi_{toy7}$, featuring additional obstacles. In $\phi_{toy6}$, the symbol *w* represents the workspace areas that are not designated as regions of interest or obstacles. For the purpose of comparative analysis, the trajectory cost in these scenarios is normalized to 1 at the 700th iteration.

Additional experiments were conducted in scenarios with increased obstacle density. Figure 10 showcases the solution paths generated by the proposed algorithm for specific LTL formulas ($\phi_{toy4}$ to $\phi_{toy7}$) over 700 iterations. In each scenario, the algorithm’s progress is depicted at various iteration milestones. The initial columns display the evolution of the search tree process through iterations, as well as the rewiring procedures, with reference to Figure 6. The final column highlights the final solution path in orange and the search tree in sky blue, without distinct representation of rewiring edges.

Table 2 showcases the outcomes of comparative experiments, similar to those detailed in Table 1. Each scenario was specifically designed with particular regions of interest and obstacle configurations, as shown in Figure 10. The increased obstacle density in these scenarios led to more frequent collision checks, thereby extending the computational time required. The experimental setup involved conducting 100 trials for each scenario, with randomized initial states and costmaps to ensure variability.

For the purposes of a comprehensive comparative analysis, the results were quantified using a relative average cost metric. The average cost recorded by the “Proposed” method at 1200 s served as the reference point (normalized to 1), allowing for a direct comparison with the performance metrics of other algorithms. The findings from these experiments validate the robust performance of the proposed algorithm, even in environments characterized by an increased presence of obstacles.

**Figure 11:**
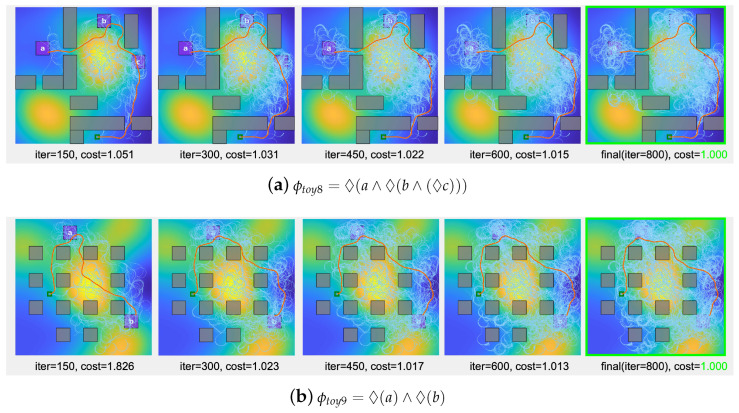

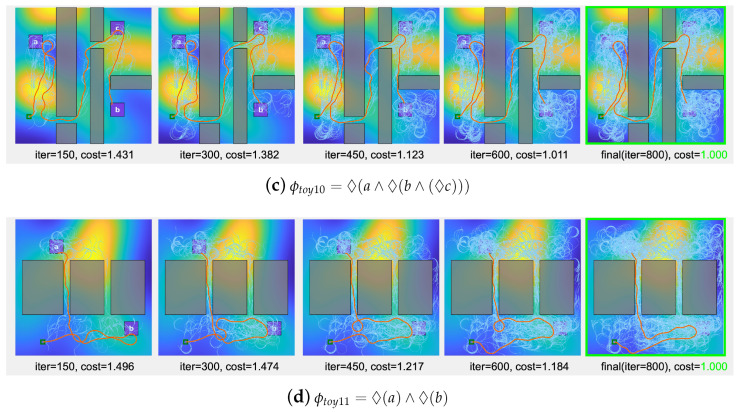
Solution paths and search tree progression for LTL formulas ϕtoy8 to ϕtoy11 are presented by the proposed algorithm under conditions of increased obstacle density. To facilitate comparative analysis, we normalize the trajectory costs to 1 at the 800th iteration, underscoring the algorithm’s efficiency even in these more intricate scenarios.

Figure 11 illustrates the adaptability of the proposed algorithm in an environment with a even higher density of obstacles, presenting an elevated challenge for path planning. The figure depicts the algorithm’s performance over 800 iterations for scenarios characterized by specific LTL formulas (from $\phi_{toy8}$ to $\phi_{toy11}$). For each scenario, the progression of the algorithm is captured at predetermined iteration milestones, offering a clear view of the search tree development (indicated in sky blue) and the evolving solution path (highlighted in red). A key observation from Figure 11 is the algorithm’s consistent ability to navigate the increased complexity and identify a viable low-cost path that complies with the given LTL mission requirements. This demonstrates the algorithm’s competence in managing densely populated obstacle spaces without compromising on the efficiency or the cost-effectiveness of the path planning process.

### 5.2. The Helsinki Traffic Accident Map

**Figure 12:**
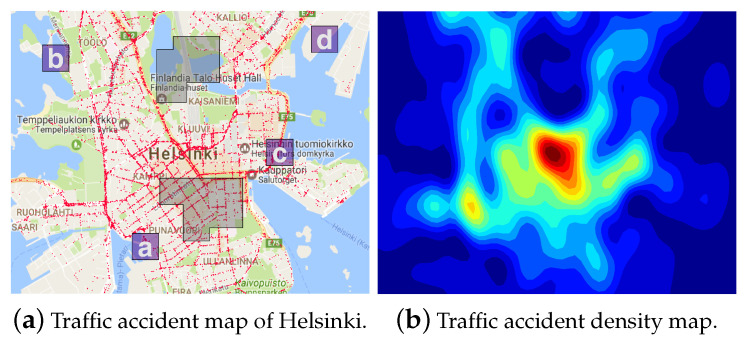
The Helsinki traffic accident map. (**a**) Areas in Helsinki where traffic accidents occurred are marked with red dots. Alphabetically labeled regions represent regions of interest, while gray zones denote obstacles. (**b**) A density map generated from traffic accident data with blue indicating low accident density and red indicating high accident density.

In the study, a navigation scenario is explored where a robot is tasked with a traffic surveillance mission in a specific area of Helsinki city, as shown in Figure 12. The scenario involves four designated regions of interest, labeled alphabetically, and obstacle zones are indicated by gray boxes. A traffic accident density map for this scenario is created using accumulated traffic accident data [53], with Gaussian process regression employed for its generation. The mission’s objective is to efficiently navigate through areas prone to traffic accidents. Consequently, zones with low accident densities are assigned lower costs, while regions with high accident densities are identified as high-cost areas.

Four distinct scenarios are tested, each with varied task specifications: Scenarios 1 and 2 are sequential missions, while Scenarios 3 and 4 are coverage missions. Their respective LTL formulas are stated as below.

- $\mathrm{Helsinki}\;\mathrm{scenario}\;1:\;\phi_{h1}=◊\left(a\wedge ◊\left(b\wedge \left(◊c\right)\right)\right)$;
- $\mathrm{Helsinki}\;\mathrm{scenario}\;2:\;\phi_{h2}=◊\left(a\wedge ◊\left(b\wedge ◊\left(c\wedge \left(◊d\right)\right)\right)\right)$;
- $\mathrm{Helsinki}\;\mathrm{scenario}\;3:\;\phi_{h3}=◊\left(a\right)\wedge ◊\left(b\right)\wedge ◊\left(c\right)$;
- $\mathrm{Helsinki}\;\mathrm{scenario}\;4:\;\phi_{h4}=◊\left(b\right)\wedge ◊\left(c\right)\wedge ◊\left(d\right)$.

Each LTL formula details the robot’s objectives. For instance, $\phi_{h1}$ implies “visit regions a,b, and *c* in sequence”, whereas $\phi_{h3}$ means “cover regions a,b, and *c*”.

**Figure 13:**
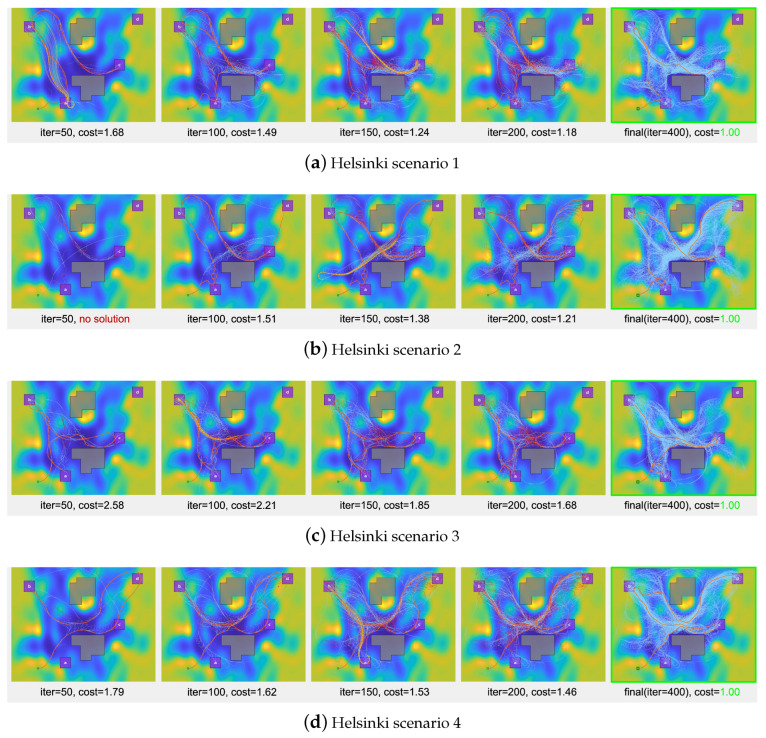
Visual representation of the tree extension method applied to the Helsinki scenarios. Annotations beneath each image convey the trajectory cost relative to the final solution’s cost (iter = 400), which is normalized to 1.

Figure 13 displays the solution paths generated by the proposed algorithm in various scenarios within 400 iterations. The algorithmic progress is shown at specific iteration counts for each scenario. The initial columns illustrate the search tree’s evolution during the iterations, as well as the application of the deep learning-based extension and rewiring procedures, with reference to Figure 6. The concluding column reveals the final solution path in orange and the search tree in sky blue, without distinct emphasis on rewiring edges. As iterations progress, the algorithm consistently produces paths that adhere to the LTL mission and traverse low-cost regions effectively.

Particularly in coverage missions, as observed in Helsinki scenarios 3 and 4, significant variations are noted in the sequence of traversing regions of interest. For instance, in Helsinki scenario 3, the initial path follows the sequence b→c→a at iteration 50, shifts to b→c→a at iteration 100, and ultimately converges to a cost-effective path of a→c→b. Similarly, in Helsinki scenario 4, the initial path sequence of c→d→b is optimized to c→b→d in the final solution. The algorithm effectively explores various plans within the search tree, leading to the identification of more cost-efficient paths that fulfill the coverage mission requirements.

### 5.3. Convergence Analysis

Our convergence analysis within a 2D environment utilized a simple dynamic model with the state equations:

$$\dot{x}=v_{x},\;\dot{y}=v_{y},$$

where (x,y) represents the position and $\left(v_{x},v_{y}\right)$ the velocity.

The performance of our algorithm was assessed using two Linear Temporal Logic (LTL) formulas:

$$\begin{array}{cc} \phi_{conv1} & =◊(a)\wedge ◊(b)\wedge ◊(c)\wedge ◊(d),\\ \phi_{conv2} & =◊(a)\wedge ◊(b)\wedge ◊(c). \end{array}$$

Here, $\phi_{conv1}$ defines a coverage mission, requiring the algorithm to visit four specified regions, while $\phi_{conv2}$ outlines a sequential mission, mandating visits to three regions in a particular order.

**Figure 14:**
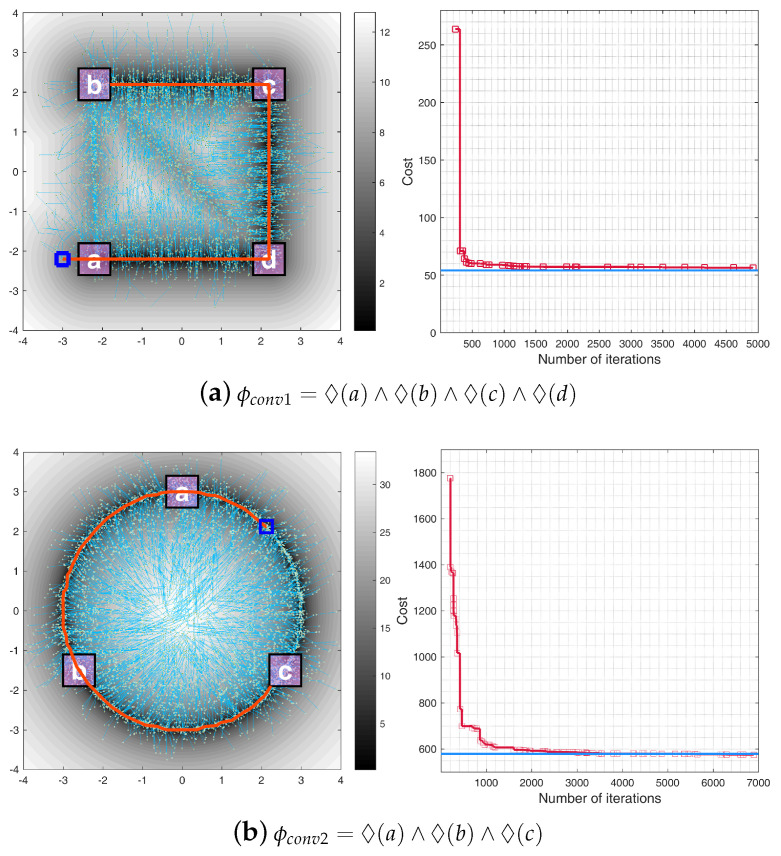
Convergence analysis for two LTL missions. The left panels show the solution paths generated by the proposed algorithm (in orange), starting from the initial state (marked by a blue square), with the search trees in sky blue. The right panels plot the trajectory costs over iterations (in red), with the optimal costs depicted for reference (in blue).

The simulation results, depicted in Figure 14, demonstrate the algorithm’s cost convergence. As the number of iterations increases, the trajectory cost approaches the optimal value, validating the asymptotic optimality of the proposed approach.

## 6. Conclusions

This paper presents a novel path planning method tailored for co-safe LTL specifications, introducing significant advancements in handling logical constraints within complex environments. A distinctive feature of this approach is its reliance on minimal discrete abstraction, eliminating the need for traditional mesh decomposition. This innovation not only streamlines the extraction of discrete plans but also aligns closely with LTL missions, significantly enhancing planning efficiency by removing the necessity for intermediate discrete plan generation during iterative processes.

Our experimental results demonstrate that this methodology markedly improves planning efficiency. The integration of a deep learning network to refine the search tree expansion process has shown to enhance the system’s performance across diverse simulation environments, providing empirical evidence of the method’s effectiveness and scalability.

Despite these advancements, we acknowledge that the approach still encounters challenges related to computational efficiency, especially in high-dimensional spaces, and limitations in the rewiring process under complex system dynamics. These limitations will be the focus of our future research, aiming to refine and optimize our method further.

Looking ahead, future research will also concentrate on overcoming the challenges of high-dimensional path planning under logical constraints with greater efficiency. Particular attention will be directed towards task-based planning for multi-joint manipulators, leveraging the learning-based strategies developed here. This focus is crucial for advancing robotic and autonomous system capabilities, enabling them to navigate complex tasks and environments more effectively.

In conclusion, the proposed path planning methodology leverages minimal discrete abstraction and deep learning-based extensions to offer a significant breakthrough in developing robust and efficient autonomous systems. These systems are well-equipped to navigate intricate environments and fulfill complex mission objectives, setting a new benchmark in the field. We are committed to addressing the noted shortcomings in future iterations of our research, enhancing both the practicality and effectiveness of our approach.

## Figures and Tables

**Table 1:** Comparative analysis of path planning algorithms across toy scenarios ($\phi_{toy1}∼\phi_{toy3}$) This table delineates the comparative performance of various path planning algorithms across three toy scenarios, labeled as $\phi_{toy1}$, $\phi_{toy2}$, and $\phi_{toy3}$. It showcases the relative average trajectory costs over 100 trials, with each algorithm’s cost normalized against the results of the “Proposed” method at 420 s. The comparison highlights the efficiency and effectiveness of the “Proposed” approach relative to other established algorithms in the field.

	Method	Time					
		120 s	180 s	240 s	300 s	360 s	420 s
$\phi_{toy1}$	Proposed	1.72	1.53	1.41	1.25	1.15	1.00
	Proposed (wo/r)	1.71	1.51	1.47	1.38	1.31	1.23
	LBPP-LTL	1.75	1.62	1.48	1.36	1.22	1.12
	CAPP-LTL	1.89	1.79	1.71	1.65	1.58	1.50
	Bahita	2.39	2.27	2.22	2.18	2.11	2.08
	McMahon	2.21	2.10	2.01	1.96	1.92	1.83
$\phi_{toy2}$	Proposed	1.45	1.37	1.29	1.19	1.11	1.00
	Proposed (wo/r)	1.43	1.36	1.28	1.22	1.19	1.15
	LBPP-LTL	1.52	1.47	1.35	1.24	1.17	1.07
	CAPP-LTL	1.65	1.55	1.47	1.41	1.32	1.20
	Bahita	2.12	2.01	1.97	1.91	1.88	1.85
	McMahon	1.89	1.82	1.78	1.73	1.71	1.69
$\phi_{toy3}$	Proposed	1.96	1.76	1.52	1.39	1.21	1.00
	Proposed (wo/r)	1.95	1.74	1.49	1.41	1.35	1.28
	LBPP-LTL	2.01	1.82	1.64	1.49	1.38	1.24
	CAPP-LTL	2.13	1.91	1.83	1.72	1.64	1.48
	Bahita	2.46	2.37	2.29	2.17	2.11	2.04
	McMahon	2.27	2.15	2.09	2.04	1.97	1.92

**Table 2:** Comparative performance of path planning algorithms in enhanced obstacle scenarios ($\phi_{toy4}∼\phi_{toy7}$) This table presents a comparative analysis of various path planning algorithms across enhanced obstacle scenarios, denoted as $\phi_{toy4}$ through $\phi_{toy7}$. It tabulates the relative average trajectory costs over 100 trials for each scenario, normalized against the results of the “Proposed” method at 1200 s.

	Method	Time					
		200 s	400 s	600 s	800 s	1000 s	1200 s
$\phi_{toy4}$	Proposed	1.72	1.56	1.38	1.25	1.18	1.00
	Proposed (wo/r)	1.80	1.65	1.47	1.38	1.30	1.23
	LBPP-LTL	1.75	1.59	1.41	1.29	1.23	1.08
	CAPP-LTL	1.83	1.70	1.51	1.42	1.35	1.28
	Bahita	2.61	2.53	2.45	2.40	2.33	2.29
	McMahon	2.35	2.28	2.21	2.13	2.05	1.99
$\phi_{toy5}$	Proposed	2.26	1.81	1.52	1.32	1.20	1.00
	Proposed (wo/r)	2.31	1.84	1.57	1.36	1.29	1.25
	LBPP-LTL	2.29	1.85	1.53	1.35	1.24	1.10
	CAPP-LTL	2.37	1.92	1.60	1.39	1.28	1.19
	Bahita	2.45	2.31	2.25	2.19	2.08	1.96
	McMahon	2.28	2.12	2.04	1.96	1.91	1.88
$\phi_{toy6}$	Proposed	1.29	1.24	1.19	1.11	1.05	1.00
	Proposed (wo/r)	1.32	1.27	1.20	1.18	1.16	1.14
	LBPP-LTL	1.33	1.25	1.21	1.14	1.09	1.06
	CAPP-LTL	1.39	1.30	1.26	1.20	1.14	1.11
	Bahita	1.68	1.62	1.59	1.52	1.49	1.45
	McMahon	1.65	1.60	1.56	1.51	1.44	1.41
$\phi_{toy7}$	Proposed	1.42	1.34	1.27	1.19	1.09	1.00
	Proposed (wo/r)	1.46	1.35	1.29	1.23	1.19	1.12
	LBPP-LTL	1.47	1.36	1.28	1.21	1.14	1.08
	CAPP-LTL	1.56	1.44	1.36	1.28	1.21	1.16
	Bahita	1.89	1.81	1.76	1.72	1.69	1.65
	McMahon	1.75	1.73	1.70	1.64	1.62	1.59

## Data Availability

The raw data supporting the conclusions of this article will be made available by the authors on request.
